# Review of the effect cultural capital and subjective socioeconomic status on life satisfaction in Iran: the mediating role of health-promoting lifestyle and the moderating role of ethnicity

**DOI:** 10.1186/s12889-023-17490-x

**Published:** 2023-12-22

**Authors:** Nader Rajabi Gilan, Jamal Mohamadi, Adel Irankhah, Mehdi Khezeli, Alireza Zangeneh

**Affiliations:** 1 Sociology Department, Faculty of Humanities and Social Sciences, University of Kurdistan, sanandaj, Iran; 2 Social Development & Health Promotion Research Center, Health Institute, Kermanshah University of Medical Sciences, Kermanshah, Iran

**Keywords:** Lifestyle, Socioeconomic inequality, Cultural capital, Health promotion, Ethnicity, Life satisfaction

## Abstract

**Introduction:**

Health-promoting lifestyle can leads to improving the quality of life, life satisfaction, well-being and reducing the burden of health care in the society. This study was carried out to investigate the mediating role of health-promoting lifestyle and moderating role of ethnicity in the effect of cultural capital and subjective socioeconomic status on life satisfaction in Iran.

**Methods:**

This cross-sectional study was conducted with 800 respondents in the cities of Kermanshah with Kurdish ethnicity and Tabriz with Azeri ethnicity. The data gathering tool was a questionnaire in five section including demographic checklist, cultural capital questionnaire (2015),Diener’s life satisfaction scale, and health-promoting lifestyle questionnaire (HPLP II), and socioeconomic status scale. Data were analyzed by SPSS and AMOS software.

**Results:**

Life satisfaction had the highest correlation with the objective dimension of cultural capital (p < 0.001 r = 0.298). The direct standardized coefficient of the path of cultural capital to health-promoting lifestyle was 0.44 (P < 0.001). Also the direct standardized coefficient of cultural capital on Life satisfaction was 0.04 that was not significant. The standard coefficient of the path of cultural capital on life satisfaction through health-promoting lifestyle was 0.27(P < 0.001). Ethnicity variable did not moderate the effect of cultural capital on life satisfaction (p > 0.05).

**Conclusion:**

The results of this study showed that paying attention to the concept of health-promoting lifestyle is a necessity to affect life satisfaction. It can play a role as a mediator for the path of cultural capital and socio-economic status on life satisfaction. This study also showed the role of ethnicity as a moderating variable in the relationship between socio-economic status and health-promoting lifestyle.

## Introduction

Non-communicable diseases (NCDs) are the main cause of chronic diseases and deaths in the world [1, 2]. The World Health Organization (WHO) has estimated that NCDs in the next decade will account for 80% of the global disease burden and half of the deaths in Asia [3, 4]. Regardless of predisposing factors, lifestyle has a major impact on morbidity and mortality during the life span [5]. Lifestyle is a specific and definable pattern of behavior that results from the interaction between personal characteristics, interaction and social relations, environmental conditions, and socio-economic status [6]. The positive consequence of a healthy lifestyle is improving the quality of life, well-being and reducing the burden of health care in society [7]. Evidence shows that adopting a healthy lifestyle is effective in preventing diseases, managing long-term health conditions, and maintaining health [8]. The importance of lifestyle is largely due to its effect on the quality of life and disease prevention, so it is necessary to promote and modify lifestyle to maintain and improve health [9]. For example, a study among Chinese students showed that by adding health-promoting lifestyle variables, the predictive power of quality of life increased significantly [10].

Life satisfaction (LS) is the main component and the most accepted measure of subjective well-being in many studies [11]. LS reflects the balance between people’s wishes and their current situation and is considered as a cognitive component of mental well-being [12]. It is associated with providing mental and physical health, increasing life expectancy, happiness, and quality of life index [13] and has an inverse relationship with the causes of death [14]. Lifestyle and LS have a significant mutual relationship, but especially LS is effective on many aspects of lifestyle and health behaviors [15]. The results of a study in Iran emphasized the positive and meaningful relationship between health-promoting lifestyle and LS in women [16].

Conceptualizing a healthy lifestyle requires something beyond individualistic paradigms and emphasis on different social structural and situational characteristics that affect people’s health-oriented behaviors [17]. Abel (2008) argues that social and economic resources play a fundamental role in the distribution of health-related consequences and emphasizes the role of culture-related factors, such as normative beliefs, knowledge and behaviors. He has also explained the role of cultural resources on the unequal distribution of health [18]. This is while the role of cultural resources compared to social and material resources on health-related outcomes and the reproduction of health inequalities have been less investigated [19]. The main body of studies on quality of life and psychological well-being have emphasized more on physical factors and less on culture and art [20].

Cultural capital (CC) is one of the non-physical factors that can have meaningful relationships with lifestyle and LS. As a sociologist, Pierre Bourdieupresented the concept of CC with an emphasis on the sociology of consumption and lifestyle [21]. Bourdieu believed that CC is formed based on tendencies, intellectual qualities and habits through education and socialization. He introduced CC in three forms: embodied, objectified, and finally institutionalized forms [22]. Abel (2008) also refers to Bourdieu’s theory and examines the role of CC in understanding the health inequalities. According to Abel, CC is the key element in changing the behavioral form of social inequality to health inequality [22]. The results of a meta-analysis in Iran showed the significant relationship between CC and lifestyle, with a moderate effect size [23]. The results of study by Kwon et al., (2018) in South Korea also showed that CC had a significant relationship with LS [24].

Another social variable that is investigated in this study is the socio-economic status (SES). SES is one of the strongest most and consistent predictors of disease and mortality [25], so that the role of socioeconomic factors on health and disease is an attractive topic worldwide [26] and can be the subject of many researches. SES is a complex phenomenon that is predicted by a wide range of variables, including financial, occupational and educational factors [25], with a fundamental role in the process of producing and imposing health inequalities [27]. Many quantitative and qualitative studies showed that a high level of SES is associated with a higher level of LS in different social groups [28–30].

Iran is a country in Southwest Asia and the Middle East region, which has diverse ethnic groups with different lifestyles. Belonging to a certain ethnicity, due to inequality in receiving health services, can strengthen certain habits and lifestyle. Therefore, it is likely that the interaction of lifestyle and habits with ethnicity creates a social atmosphere in which health-related behaviors are formed and strengthened. Some studies indicate that few studies have been conducted regarding the relationship between ethnicity and health preventive behaviors [31] and there is a significant knowledge gap in this field [32]. The results of a study showed that there are differences between two ethnic groups (American and African) in healthy eating, regular exercise, and seeing a doctor. These differences emphasize the necessity of creating health promotion messages in accordance with cultural background in order to maximize preventive health behaviors in different ethnic groups [31]. Also, the literature shows that life satisfaction is also affected by ethnicity [33, 34]. For example, a study in Iran showed that Lur ethnicity had a lower level of LS than Fars and Azeri ethnicities in Fars province population [35]. The results of another study also showed that the mental well-being and satisfaction with life in the Kurds and Lur ethnicity was lower than that of Fars and Azeris [36]. The most important path explained for this difference is through social inequalities related to ethnicity including income, employment, health and social resources [33, 37]. Ethnic minorities make up almost half of Iran’s population, and the rest are Persians. Azeris and Kurds, as two large ethnic groups in Iran (with about 32% of Iran’s population), which are culturally, linguistically, and religiously different, were sample of this study [38].

The first goal of this study was to investigate the effect of CC dimensions and subjective socio-economic status (SSS) and health-promoting lifestyle on LS (as an outcome variable). The second goal of the article was to evaluate the role of health-promoting lifestyle as a mediating variable and role of ethnicity as a moderating variable in relation to the main research variables in Iran.

## Methods

### Study design and participants

This cross-sectional study was conducted with 792 people in 2021, in the west and northwest of Iran. The statistical population of the study was women and men aged 18 to 64 from Kermanshah city as the capital of Kermanshah province in the west of Iran with a population of 1,153,300 and also Tabriz city as the capital of East Azarbaijan province in the northwest of Iran with a population of 1,643,960 people.

Cluster sampling method was used in this study. For this purpose two municipality district was considered as clusters in each city. Then, two neighborhoods were randomly selected from each district. Finally, participants were selected in each neighborhood using convenience sampling method. Inclusion criteria were: having 18 to 64 years old, consent to participate in the study, and lack of physical disability and acute mental illness. Incomplete questionnaires were excluded from analysis. The sample size was estimated to be 400 people with Cochran’s formula, which was finally increased to 800 peoplewith design effect equal to 2.As a matter of fact, in total, 792 people (Kermanshah 398 sample and Tabriz 394 sample) completed the data gathering tools, demonstrating a response rate of 99%.

### Measurements

A five sections questionnaire was used to collect data. The first part included questions about demographic and contextual variables such as age, sex, marital status, education level, job status, and chronic disease. The second part was cultural capital questionnaire (CCQ),introduced by Hasanpour and Ghasemi (2015). CCQ has 21 items to measuring the three types of cultural capital including: objectified dimension with 11 items, embodied dimension with 7 items, and institutional dimension with 3 items. A higher score on the CCQ indicates greater CC. The result of the model fitting study indicated that questionnaire had the acceptable qualities in the four criteria of internal consistency, index reliability, convergent validity and divergent validity [21]. The third part was the questionnaire of SSS by which respondents rated their SSS. The subjective evaluation of SES is the self-conceiving of the individual’s position in the social structure This scale evaluates perception of individuals about job, education, and wealth dimensions on a 10-point ladder, in which the higher score indicated the better perception about SSS [39, 40].

The fourth part was the Satisfaction with Life Scale (SWLS)developed by Diner et al., (1985) [41]. This scale has five items, each rated on a five-point Likert scale (1 = completely disagree to 5 = completely agree). The overall score of the scale varies between 5 and 35. The Persian version of SWLS has been validated by Vahedi and Eskandari (2010) with an acceptable reliability using Cronbach’s alpha. Using concurrent validity assessment, there was a significant correlation between SWLS and the four subscales of WHO Quality of Life questionnaire including mental health, physical health, social relationships, and environmental health [42]. The final part was Health-Promoting Lifestyle Profile II (HPLP-II). This tool has been widely used in research and its appropriate validity and reliability have been reported in different populations [43, 44]. This questionnaire has 52 questions covering six dimensions including health responsibility (HR, 9 items), physical activity (PA, 8 items), nutrition (N, 9 items), spiritual growth (SG, 9 items), interpersonal relations (IR, 9 items), and stress management (SM, 8 items). Participants rated each item on a four-point format (1 = never, 2 = sometimes, 3 = often, and 4 = routinely). Overall, the score for health-promoting lifestyle and dimensions is calculated using the mean of responses for all 52 items and for each subscale (eight or nine items). The lowest and highest total score is 52 and 208, respectively. The validity and reliability of the Persian version of HPLP-II has been confirmed by Mohammadi Zaidi et al [45].

### Ethical statement

This study received the ethics approval from the Research Ethics Committee of Kermanshah University of Medical Sciences (No. IR.KUMS.REC.1397.070). Written informed consent form was obtained from all of the participants.

### Data collection and statistical analysis

SPSS software (descriptive indices including mean and standard deviation, and Pearson correlation) and AMOS software (structural equation model- SEM) were used for data analysis. After ensuring the validity of the measurement model based on the first-order confirmatory factor analysis, all components of the research were imputed (in this method, all measurement error coefficients are calculated) and the direct and indirect path coefficients between hidden variables were calculated in a structural model.

## Result

This study was conducted with the participation of 792 people, 398 Kurds (50.3%) and 394 Azaries (49.7%). Women and men equally formed half of the sample. The mean age of the participants was 34.48 ± 11.91 years. Most of the respondents were married (56.2%) or single (40.2%). The employment status showed that 41.4% were employed and 30.6% were unemployed. The results also showed that 11.5% of participants had one or more types of chronic diseases. More details on demographic information by ethnicity are provided in Table 1.

**Table 1:** Demographic characteristic based in ethnicity (Kurdish and Azeri)

Variables	Category	Kurds N (%)	Azeris N (%)
Sex	Male	200(50.3)	196(49.7)
	Female	198(49.7)	198(50.3)
	Total	398(100.0)	394(100.0)
Married	Single	142(35.8)	175(44.8)
	Married	241(60.7)	204(52.1)
	Widow	14(3.5)	12(3.3)
	Total	397(100.0)	363(100.0)
Job	Employed	174(44.3)	154(42.4)
	Housekeeper	94(23.6)	65(17.9)
	Unemployment	110(28.0)	132(36.4)
	Retirement	15(3.8)	15(3.8)
	Total	393(100.0)	363(100.0)
Chronic disease	Yes	360(90.5)	337(86.4)
	No	38(9.5)	53(13.6)
	Total	398(100.0)	390(100.0)
Total		398 (50.3)	394 (49.7)

Table 2 shows the comparison of the mean scores of the main variables based on demographic information. The mean score of CC was significantly higher in women than in men and lower in people with chronic diseases than healthy people. Health-promoting lifestyle was significantly higher in healthy people than people with chronic diseases.

**Table 2:** Descriptive statistics of the demographic variables of the participant (n = 792)

Variable	Category	N (%)	Total cultural capital		health promoting lifestyle		Life satisfaction	
			Mean ± SD	p-value	Mean ± SD	p-value	Mean ± SD	p-value
Sex	Male	396(50)	17.51 ± 12.35	0.002	120.18 ± 20.67	0.189	13.40 ± 4.30	0.261
	Female	396(50)	20.27 ± 12.60		122.12 ± 19.61		13.74 ± 4.17	
Married	Single	317(40.0)	21.90 ± 12.68	0.130	122.10 ± 20.43	0.799	13.85 ± 4.29	0.950
	Married	445(56.2)	17.01 ± 12.02		121.17 ± 20.87		13.51 ± 4.23	
	Widow	26(3.8)	16.05 ± 13.17		115.26 ± 18.01		12.19 ± 3.33	
Job	Employed	328(41.4)	20.16 ± 13.75	0.797	122.84 ± 21.72	0.863	13.66 ± 4.13	0.941
	Housekeeper	159(20.1)	14.47 ± 9.33		119.13 ± 19.14		13.01 ± 4.18	
	unemployment	242(30.6)	19.43 ± 11.95		119.34 ± 20.89		13.45 ± 4.36	
	Retirement	27(3.4)	17.27 ± 10.76		122.76 ± 18.43		14.14 ± 4.08	
Chronic disease	Yes	91(11.5)	15.45 ± 10.32	0.001	117.10 ± 20.92	0.050	13.16 ± 4.22	0.026
	No	697(88.5)	19.34 ± 12.76		121.71 ± 20.63		13.62 ± 4.23	
Total participant		792(100.0)	18.90 ± 12.54		121.17 ± 20.67		13.58 ± 4.24	

Descriptive analysis showed that the mean score of health promoting lifestyle and LS was higher in Kurds than in Azeris, althoughthis difference was not significant (p = 0.753; p = 0.154).The mean score of SSSin Azeri speakers was higher than Kurdish speakersand this difference was significant (p = 0.001).(Table 3).

**Table 3:** Comparing descriptive statistics and main research variables according to Kurdish (Kermanshah) and Azeri (Tabriz) ethnicities

Variable	Dimension	Kermanshah (Kurdish) Mean ± SD	Tabriz (Azeri) Mean ± SD	P- value
cultural capital	Objectified	6.61 ± 6.67	8.70 ± 6.24	0.001
	Institutional	3.01 ± 3.00	3.00 ± 2.62	0.943
	Embodied	7.50 ± 6.07	8.97 ± 6.07	0.001
	Total	17.13 ± 12.98	20.68 ± 11.82	0.001
health promoting lifestyle	Health Responsibility	21.63 ± 4.93	21.32 ± 3.90	0.326
	Physical Activity	19.55 ± 4.02	19.57 ± 3.56	0.963
	Nutrition	20.08 ± 4.23	19.97 ± 3.74	0.678
	Spiritual Growth	21.33 ± 4.06	20.04 ± 3.77	0.001
	Interpersonal Relations	20.42 ± 4.08	20.93 ± 3.73	0.066
	Stress Management	19.19 ± 3.78	18.27 ± 3.50	0.001
	Total	122.22 ± 22.00	120.12 ± 19.21	0.154
Life satisfaction		13.63 ± 4.29	13.53 ± 4.19	0.753
subjective socioeconomic status		4.24 ± 1.93	5.18 ± 1.81	0.001

Pearson’s correlation analysis showed a significant relationship between LS and SSS(r = 0.370 p < 0.001). Among the dimensions of health promoting lifestyle, interpersonal relationships had the highest positive correlation with LS(r = 0.549, p < 0.001). LS had also the highest correlation with the objective dimension of CC (r = 0.298, p < 0.001). (Table 4)

**Table 4:** Correlation matrix of the main research variables

Variables	Dimensions	Cultural capital			Life satisfaction	Subjective socioeconomic status
		Objectified	Institutional	Embodied		
Health promoting lifestyle	Health Responsibility	0.391**	0.212**	0.323**	0.548**	0.313**
	Physical Activity	0.358**	0.136**	0.179**	0.541**	0.260**
	Nutrition	0.410**	0.150**	0.250**	0.504**	0.288**
	Spiritual Growth	0.364**	0.173**	0.262**	0.465**	0.206**
	Interpersonal Relations	0.417**	0.164**	0.292**	0.549**	0.316**
	Stress Management	0.320**	0.160**	0.259**	0.460**	0.196**
Life satisfaction		0.298**	0.087*	0.206**	1	0.370**
Subjective socioeconomic status		0.317**	0.170**	0.283**	0.370**	1

*Significant at 0.05 level

**Significant at 0.001 level

Confirmatory factor analysis was used to check the extracted factors, according to which the results of goodness of fit indices of the model are shown in Table 5. The results of Table 5 show that the proposed model has a good fit.

**Table 5:** Direct and indirect coefficients of the main variables of the study

Paths	Direct Coefficients			Indirect Coefficient		
	B	β	P-value	B	β	P-value
CC → H-P-L-F	0.38	0.44	0.001	-	-	-
SES → H-P-L-F	0.12	0.15	0.001	-	-	-
H-P-L-F→ LS	0.60	0.60	0.001	-	-	-
CC →LS	-0.007	-0.04	0.328	0.04	0.27	0.001
SES $\to$ LS	0.089	0.22	0.001	0.04	0.09	0.001

IFI = 0.96, CFI = 0.96, RFI = 0.94, NFI = 0.95, AGFI = 0.91, GFI = 0.94, RMSEA = 0.34, CMIN = 2.77

The results of Table 5 show that the direct standard coefficient of the path of CC to lifestyle was 0.44, which is significant. Also, the direct standard coefficient of the lifestyle path on LS was 0.60, showing asignificant effect. On the other hand, the direct standard coefficient of CC on LS was 0.04, which was not significant at the P < 0.001 level.

Also, the results showed that the standard coefficient of cultural capital’s effect on LS through lifestyle was 0.27, which was a significant effect. This means that lifestyle has an indirect or mediating role in the relationship between CC and LS. Also, the indirect role of health-promoting lifestyle in the relationship between SSS and LS was significant. In other words, health-promoting lifestyle has a mediating role in the relationships of CC and SSSwith LS (Table 5).

**Fig. 1:**
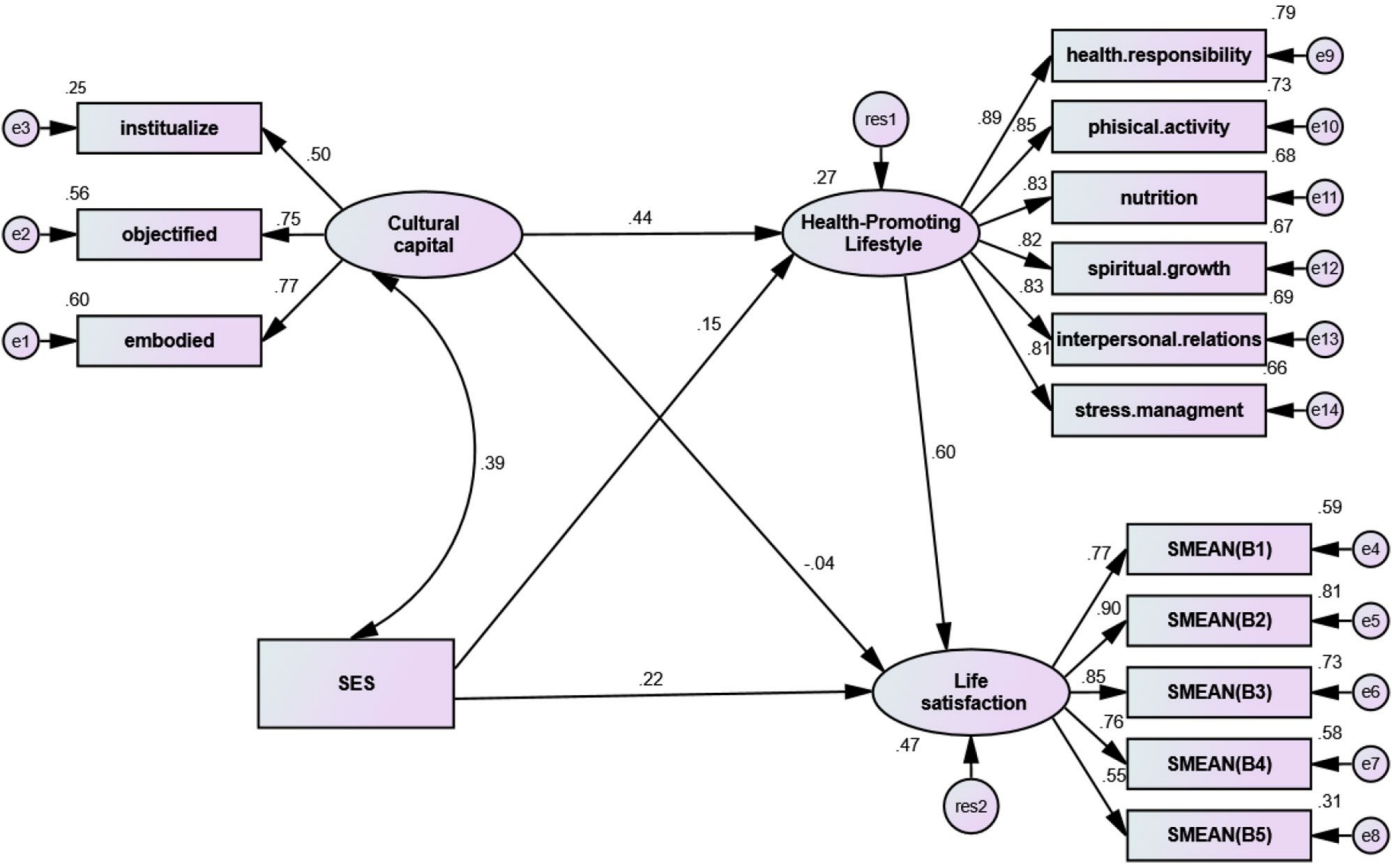
Structural equation model (SEM) in the total sample

As shown in Fig. 1, the results obtained from the final standardized model showed that the variables of CC and SSShave explained a total of 27% of health-promoting lifestyle changes. Also, the variables of CC, health-promoting lifestyle and SSSexplained a total of 47% of the changes in the variable of LS.

As shown in Table 6, in Kermanshah metropolis, CC and SSS had an impact on health-promoting lifestyle (p < 0.001). Health-promoting lifestyle and SSS had a significant effect on LS (p < 0.001). Also, there is a significant relationship between CC and LS (p < 0.027).On the other hand, the findings of the study in Tabriz metropolis showed that health-promoting lifestyle was significantly affected by CC, but had no significant relationship with subjective socioeconomic status. Health-promoting lifestyle and SES had a significant effect on LS, while CC did not affect LS (Table 6).

**Table 6:** comparison of the moderating effect of ethnicity (Kurdish and Azeri) on the relationship between mainvariable

Paths			Kurdish			Azeri			Z-score
			B	β	P-value	B	β	P-value	
Health-promoting life style	<---	Cultural capital	0.381	0.39	0.001	0.375	0.49	0.001	-0.079
Health-promoting life style	<---	SES	0.605	0.27	0.001	0.141	0.07	0.14	-3.023**
Life satisfaction	<---	Cultural capital	-0.02	-0.13	0.027	0.01	0.59	0.384	2.083*
Life satisfaction	<---	Health-promoting life style	0.093	0.57	0.001	0.141	0.05	0.001	2.757**
Life satisfaction	<---	SES	0.131	0.35	0.001	0.065	0.14	0.001	-2.447*

*Significant at 0.05 level

**Significant at 0.001 level

Table 6 also shows that ethnicity had a moderating effect on the relationship between CC, SSS and LS. The findings indicated that the effect of CC on health- promoting lifestyle was not significant according to ethnicity. In other words, ethnicity does not moderate the path between CC and health- promoting lifestyle (Z-score = − 0.079).

## Discussion

This study was conducted aimed to investigate the effect of CC and SSS on the health-promoting life style and LS in the west and northwest of Iran (Kermanshah and Tabriz).In the present study, CC had a direct effect on health-promoting lifestyle. However, the results of the study conducted in Iran showed that CC had an indirect effect on health-promoting lifestyle [46], inconsistent with this study. The results of the study by Gagne et al., (2015) also showed that CC may lead to better understanding of the social resources through which people are guided towards healthy behaviors [47]. Ghaderi et al., (2016) found that the dimensions of CC related to health have an explanatory effect of 62% on healthy lifestyle, and the contribution of embodied CC related to health in this prediction was more than others [48]. Mohammadi et al., (2013) in Iran and among the Kurdish ethnic groups showed that CC and its dimensions had positive and meaningful relationships with healthy lifestyle, consistent with the results of this study [49]. Hashemi et al., (2018) also showed that CC can affect health through health behaviors in such a way that people with a higher level of education are likely to have healthier eating habits, more physical activity, and less tendency to smoke and drink alcohol. In addition, CC is probably an important component in transforming social inequality into health inequality through specific lifestyles in different social classes [50]. These findings support Bourdieu and Cockerham’s point of view that lifestyle, and in a way health-oriented lifestyle, is influenced by the amount and structure of CC [51]. Bourdieu’s comments have addressed the healthy lifestyle in three areas: First, the concept of distance from need as the origin of class differences in lifestyles. Second, identifying the role of habitus in the production and reproduction of lifestyles, and thirdly, emphasizing this role, or in other words, going beyond Weber and Giddens to show how the structure or “life opportunities” determine lifestyle choices [52]. In general, the results of this study regarding the impact of CC on health-related lifestyles are far from individual-centered lifestyle analyzes and are in line with the study of Farrell et al., (2008) who pointed out the macro-structural causes in the society such as the health care system, health inequalities, and community and social participation [53].

The results of the present study showed that CC had no effect on LS, similar to the study by Steiner et al., (2015) where they mentioned that this situation in Europe is probably caused by dissatisfaction with the high level of public expenses, transportation disturbances and the increase in housing prices [54].Probably due to the economic and social crises that Iran is facing, the economic conditions have also affected our study sample. Therefore, we suggest that this situation be investigated in future studies. However, the results of the study by Jagodzinski et al., (2010) contrary to the results of our study showed the effect of cultural variables on LS in Asia and Europe [55]. The results of the study of Kwon et al., (2018) in Korea also showed that CC has a significant relationshipwith LS [24].It can be stated that LS as a function of wishes and expectations is influenced by micro and macro level variables. In this regard, CC under certain conditions may improves the opportunity structure for the individual and increase LS [55]. It seems that the wideness of the concept of LS and its dimensions (satisfaction with job, income and marital relations) caused that in this study, the variable of CC cannot be a suitable explanation for LS. In the present study, it seems that socioeconomic status are stronger in explaining LS than CC. Probably, the role of CC in environments where material values have increased and success depends on economic capital has decreased and may even be ineffective.

Another result of this study was the effect of subjective socio-economic status on LS and health-promoting lifestyle, where the effect of SSS on LS was greater than health-promoting lifestyle. The results of the studies conducted in Iran have also shown that the SSS has been the strongest predictor of health in the Iranian population [56]. RajabiGilan et al., (2021) in a study in the urban population in Iran showed thatSSS have a significant effect on respondents’ LS, which is in line with the results of this study [57].In this regard, the results of the study by Wang et al., (2018) in China also indicated that the socio-economic status of a person during childhood and adulthood were associated with the health of the elderly. Employment status, financial status and residential conditions have been among the variables that have influenced the health-related lifestyle [58]. In addition, the results of study by Abbott and Sapsford (2006), also showed that human capital, material conditions, satisfaction with income, and family facilities were associated with LS. They argued that the low socio-economic status causes a person to experience pessimism, despair, and a feeling of helplessness, and subsequently leads to a decrease in LS. Improving socio-economic status by influencing the choice of healthy lifestyles can reduce the incidence of diseases and ultimately mortality [59].

Another object of this study was to investigate the mediating role of health-promoting lifestyle. The results showed that health promoting lifestyle mediates the relationship between CC and socio-economic status with LS. KochaniEsfahani et al., (2018) have emphasized the role of health-promoting lifestyle in the relationship between CC and public health [60]. Kamalinejad et al., (2020) have also shown the role of health-promoting lifestyle in the relationship between coping strategies and quality of life in students [61]. In addition, Seo et al., (2018) in Korean students [62], Hu et al., (2022) in the elderly [63], and Xue et al., (2021) in seniors [64], emphasized the mediating role of health-promoting lifestyle.Although in this study, CC did not have a direct and meaningful effect on LS, but with the presence of health-promoting lifestyle, this effect becomes significant indirectly, which shows that lifestyle is affected by the amount and structure of CC.It can be claimed that the position of health-oriented behaviors in the lifestyle will have more effects on LS, quality of life.According to the findings of this study and regarding the explanation of LS, it can be said that CC in its Bourdieuian meaning has far less explanatory power than Abel’s point of view for explaining LS. Abel (2008) by proposing the concept of CC related to health, emphasizes those cultural resources accessible that are most relevant in the field of health and wellness [22]. In other words, CC alone is not able to explain LS, but the combination of lifestyle with it causes more power to explain LS.

Another finding of this study was the moderating effect of ethnicity on the relationship between the main research variables. Various studies have emphasized the role of ethnicity in the relationship between some psychological and social variables [65–69],which is consistent with some findings of this study. Growing evidence suggests that racial/ethnic disparities in mortality and health outcomes are related to socioeconomic resources, largely based on the health experiences of blacks and whites, with much less evidence in other ethnicities/races [70].The role of socioeconomic factors as a cause of racial/ethnic health disparities is complex. It has been suggested that socioeconomic differences often “do not explain” all of the health differences between African Americans and non-Hispanic whites, and that black-white differences in health remain after controlling for socioeconomic conditions [70]. In the present study, the findings showed that ethnicity does not moderate the relationship between CC and health-promoting lifestyle.This shows that the relationship between these two variables is independent of ethnic factors and contexts and is mostly caused by the volume and structure of cultural resources. On the other hand, in relation to other variables, ethnicity has a moderating role.

## Conclusions

This study showed that the health-promoting lifestyle had a mediating role in the relationship between CC and the subjective socioeconomic status, and was able to make meaningful the relationship between CC and LS. Also, ethnicity as a moderating variable, except for the relationship between CC and health-promoting lifestyle, had a moderating effect on the relationships of all the main variables. One of the limitations of this study was its cross-sectional design. Also, this study was conducted in two Iranian ethnic groups, Azerisand Kurds, and due to financial considerations and geographical conditions, other ethnic groups such as Baloch were not studied in this study. Therefore, due to the lack of studies in the social and cultural field that deal with the moderating effect of ethnicity in Iran, it is suggested that the effect of ethnicity be considered in future studies.

## Data Availability

The data sets used and analyzed in this study are available from the corresponding author on reasonable request.

## Abbreviations

CC: Cultural capital
CCQ: cultural capital questionnaire
HPLP-II: Health-Promoting Lifestyle Profile II
LS: life satisfaction
NCDs: non-communicable diseases
SES: socio-economic status
SEM: structural equation model
SSS: subjective socioeconomic status
SWLS: Satisfaction with Life Scale
WHO: World Health Organization
